# Water consumption in 0-6-month-old healthy infants and effective factors: A systematic review

**DOI:** 10.7705/biomedica.6745

**Published:** 2023-06-30

**Authors:** Özge Karakaya Suzan, Özge Kaya, Tuğçe Kolukısa, Oğuz Koyuncu, Seda Tecik, Nursan Çınar

**Affiliations:** 1 Department of Nursing, Faculty of Health Sciences, Sakarya University, Sakarya, Turkey Sakarya University Sakarya University Sakarya Turkey; 2 Institute of Health Sciences, Nursing Doctorate Program, Sakarya University, Sakarya, Turkey Sakarya University Sakarya University Sakarya Turkey

**Keywords:** Drinking, drinking water, infant, breastfeeding, systematic review., ingestión de líquidos, agua potable, lactancia materna, revisión sistemática.

## Abstract

**Introduction:**

Early introduction of fluids and water affects the duration of breastfeeding, the infant immune system, and possibly causes infants to consume less breast milk, which may, in turn, affect their nutritional and immune status.

**Objective:**

This study was carried out to determine water consumption in 0-6-month-old infants and the factors affecting this consumption.

**Materials and methods:**

A literature review was conducted in seven electronic databases (Medline, Web of Science, PubMed, ScienceDirect, Scopus, Cochrane Library, and TÜBITAK) for studies published until April 25, 2022, using the keywords: drinking water, infant, and breastfeeding.

**Results:**

The systematic review included 13 studies. Five studies were crosssectional, three were descriptive and quasi-experimental, and the others were case-control and cohort studies. It was reported in the examined studies that 86.2% of the infants were around 6 weeks old, 44 % of the infants were 1 month old, 77% were 3 months old, 2.5% were 4 months old, and 2.5 to 85% of the infants were around 6 months old when they first consumed water. The prominent reasons for making the infants drink water are the thought that they need it and cultural reasons.

**Conclusions:**

The exclusive breastfeeding of 0-6-month-old infants is the recommendation of reliable health authorities. Nurses play a key role in implementing this practice. In this systematic review, it was seen that families gave their infants water at varying rates in the 0-6-month period, and the factors affecting this situation were revealed. If nurses determine which factors affect families in terms of the early introduction of fluids, they could be able to plan the necessary education and interventions.

## Resumen

**Introducción:**

La introducción temprana de líquidos y agua afecta la duración de la lactancia, el sistema inmune del lactante y posiblemente hace que los lactantes consuman menos leche materna, lo que a su vez puede afectar su estado nutricional e inmunitario.

**Objetivo:**

Este estudio se realizó para determinar el consumo de agua en bebés de cero a seis meses y los factores que inciden en este consumo.

**Materiales y métodos:**

se hizo una revisión de la literatura en siete bases de dato electrónicas (Medline, Web of Science, PubMed, ScienceDirect, Scopus, Cochrane Library y TÜBITAK) para estudios publicados hasta el 25 de abril de 2022, utilizando las palabras clave: agua potable, lactante y lactancia.

**Resultados:**

La revisión sistemática incluyó 13 estudios. Cinco fueron estudios transversales, tres fueron descriptivos, cuasiexperimentales y los restantes fueron estudios de casos y controles, y de cohortes. En los estudios examinados se informó que le dieron agua al 86,2 % de los lactantes de seis semanas, al 44 % de los lactantes de un mes, al 77 % de los lactantes de tres meses, al 2,5 % de los lactantes de cuatro meses y al 2,5 % a 85 % de los lactantes de seis meses. Las razones principales para dar agua a los bebés son la idea de que los bebés necesitan agua y razones culturales.

**Conclusiones:**

La lactancia materna exclusiva es la recomendación de las autoridades sanitarias para los bebés de cero a seis meses. Las enfermeras juegan un papel clave en la implementación de esta práctica. En esta revisión sistemática se observó que las familias daban agua a sus bebés en proporciones variables durante el período de cero a seis meses y se revelaron los factores que inciden en esta situación. Si las enfermeras determinan qué factores afectan a las familias en cuanto a la introducción temprana de líquidos, podrían planificar las medidas educativas y las intervenciones necesarias.

Nutrition in the first stage of life is very important for growth and development ^1^. Infants between 0 and 6 months of age are in a critical period of their growth regarding sustainable development goals ^2^^,^^3^. The World Health Organization (WHO) and the United Nations International Children’s Emergency Fund (UNICEF) recommend starting breastfeeding within the first hour after birth, exclusively breastfeeding for a minimum of six months and for two years or longer with the addition of appropriate complementary foods at the end of the 6-month of life ^4^^,^^5^.

Only 38% of 0-6-month-old babies in the world are exclusively fed with human milk ^6^. Exclusive breastfeeding rates in infants younger than six months have been reported as 1% in the United Kingdom, 12% in Azerbaijan, 16% in Afghanistan, 16.4% in the United States, 30% in South Africa, 51% in China, and 53% in Pakistan and Egypt ^7^^-^^9^. According to the 2018 Turkey Demographic and Health Survey (TDHS) report, 41% of infants younger than six months are exclusively breastfed. This rate rapidly decreases from 59 % in babies aged 0-1 months to 45 % in babies aged 2-3 months and to 14 in babies aged 4-5 months. Additionally, it was stated that 23% of infants younger than 6 months old are given products like cow’s milk, and 12% are given other food in addition to human milk ^10^.

Factors such as late initiation of breastfeeding, early or late transition to complementary feeding, giving water or sweetened water to the baby as the first food, using bottles and pacifiers, the education and employment status of the mother, lack of information about breastfeeding, and lack of health personnel/family support, result in inadequate levels of breastfeeding and human milk intake ^8^^,^^9^^,^^11^^-^^14^. The beneficial effects of breast milk come from its uniqueness and infant-specific content ^15^. Breastmilk meets all babies’ needs in their first six months; 50% of these needs between the 6th and the 12th month, and 30% of these needs after the 12th month ^16^. Families may feel the need to give their babies water due to their concerns that their babies will be dehydrated, but because water has essentially no calories, and it increases the feeling of satiety in the infant, it can lead to lower consumption of breastmilk by the infant, insufficient calorie intake, and thus, elevated bilirubin levels ^17^. Nevertheless, human milk consists of macronutrients such as proteins, carbohydrates, and fats; and micronutrients such as vitamins and minerals; also it contains 85% water ^18^.

Early introduction of fluids affects the duration of breastfeeding, the infant’s immune system, infant morbidity and mortality, mother-infant attachment, infant growth and development, length of hospital stay, physiological jaundice, maternal self-confidence, and the breastfeeding process. These possibly cause infants to consume less human milk, which may, in turn, affect their nutritional and immune status ^17^^,^^19^.

Parents may think that normal fluid loss and dehydration in babies caused by inadequate feeding can be prevented by fluid supplementation; however, the replacement of milk with water will reduce the calorie intake of the baby, leading to more weight loss in the early postpartum period. Due to the reduced calorie intake caused by malnutrition, the bilirubin levels will not decrease ^20^^-^^24^. Therefore, the introduction of water to infants younger than 6 months old is problematic as it may replace the total amount of nutrient- rich breast milk or infant formula to be consumed by the infant ^25^. While significant evidence in the literature report that healthy infants do not require solid or liquid food for the first six months other than human milk, infants with special conditions may need supplemental foods or liquids ^17^. Despite all evidence, the rates of healthy 0-6-month-old infants given water have been reported as 21%, 24.2%, 62.6%, and 70.7% ^25^^-^^28^.

Nurses and other health professionals are in an authorial position to guide parents to exclusively breastfeed their babies for the first six months ^29^. As a result of the care they provide with their consultancy roles, factors affecting the water consumption of babies in the first six months can be determined and water consumption rates can be reduced as a result of education to be given to parents. To our knowledge, there is no systematic review examining the consumption of water in addition to human milk in the first six months and the factors affecting it. This review focuses on healthy infants and does not address special cases.

This study was carried out to determine water consumption in 0-6-month- old babies and the factors affecting this consumption.

## Materials and methods

Before initiating the review, the protocol was registered in the International Prospective Register of Systematic Reviews (PROSPERO) (CRD 42022322840). This study was based on the PRISMA-P guideline, which is used to guide authors in improving the submission of systematic reviews ^30^. Studies published between 2002 and 2022 investigating water consumption in healthy 0-6-month-old babies and the factors affecting water consumption were searched between March 17 and April 25, 2022, and the reviews of the articles were completed by July, 2022.

### Eligibility criteria

Studies were selected according to criteria regarding Population, Intervention, Comparator, Outcome(s) of interest, and Study design (PICOS framework) ^31^. These were detailed as follows:

Type of population: 0-6-month-old healthy infants

Type of exposure: Water consumption

Type of comparators: None

Type of outcome measurements: Prevalence of water given to 0-6-month- old babies and factors affecting the introduction of water

Study design: Cross-sectional studies, case-control studies, cohort studies, quasi-experimental studies, randomized controlled trials, and descriptive/case series studies

### Inclusion criteria

The inclusion criteria for the systematic review:

- samples consisting of healthy 0-6-month-old babies;
- use of quantitative data analysis methods;
- samples consisting of healthy babies;
- studies published in Turkish or English between 2002 and 2022;
- accessible full-text; and,
- randomized controlled, experimental-quasi-experimental, cohort, cross- sectional descriptive, and case-control studies.

### Exclusion criteria

Studies with qualitative design or including infants diagnosed with a chronic health problem or had special nutritional needs; expert opinions, systematic reviews, unpublished thesis studies, and studies whose full text was not available were not included in the study.

### Research strategy

Keywords were created based on the research question. For the keywords in English, MeSH (Medical Subject Headings) was used. The search was conducted on the Medline, Web of Science, PubMed, ScienceDirect, Scopus, the Cochrane Library, and TÜBÍTAK Ulakbim databases by using the following keyword string: (“drinking water”) AND (infant OR baby OR infancy) AND (breastfeeding).

### Selection of studies

**Figure 1.**
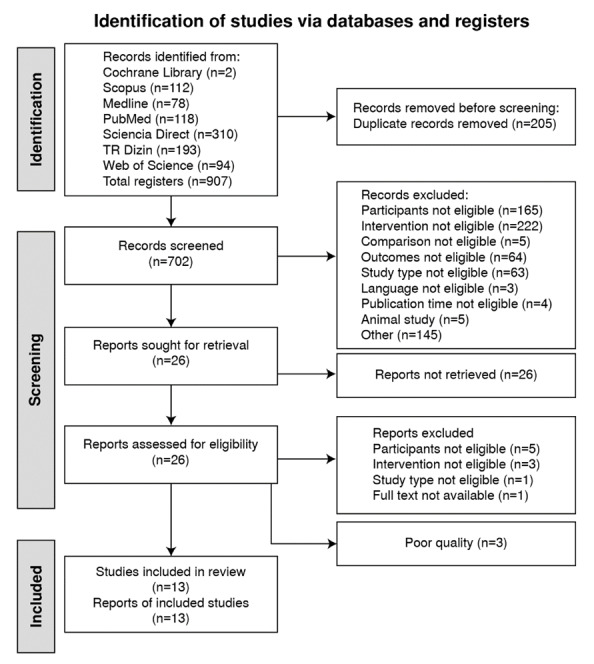
Flow diagram of the selected studies

The selection process consisted of three steps. These steps included searching in databases and evaluating the titles, abstracts, and full texts of the studies product of the searches. The searches in the databases were performed by two independent authors, and then, the found studies were evaluated in terms of suitability based on their titles and abstracts. Disagreements on the evaluation of the studies, if any, were resolved by the involvement of a third researcher. As a result of the evaluation, a consensus was reached among the researchers. Studies that met the inclusion criteria according to their abstracts were stored in an EndNote library (EndNote software X9) with their full texts. Studies selected for the systematic review are reported in a PRISMA flowchart (figure 1).

A total of 13 studies were included in the systematic review. The systematic review was conducted separately by two researchers). In a later comparison, the agreement rate between the inclusion criteria of the researchers was found to be 100% (figure 1).

### Data extraction and management

A data coding form was used to collect statistical data and study characteristics (e.g., year of publication, country, method, sample, measurement tool, and type of publication). The title, author, date of publication, country, study type, design, sample size, demographic characteristics of the infants and the family, the feeding style of the infant, the properties of the water given to the infant, the time (0-6 months old) of the first water intake by the infant, the frequency and the amount (ml/day) of ingested water, and the reasons for giving water were coded for each study.

The reliability of the data was achieved by comparing the coding processes of the first and second researchers. Disagreements about coding, if any, were resolved through discussion with the involvement of another researcher. Another researcher independently checked the extracted data for accuracy and completeness.

### Assessment of risk of bias

A bias risk assessment was made, by two reviewers at independent times, using the Joanna Briggs Institution (JBI) guidelines. These guidelines included the Checklist for Analytical Cross-Sectional Studies, the MAStARI critical appraisal tools for descriptive studies ^32^, the Checklist for Quasi- Experimental Studies, the Checklist for Case-Control Studies, and the Checklist for Cohort Studies. The scale items were scored as “Yes” = 1 point, “Unclear” and “Not Applicable” = 0 points. A high total score indicated a high- quality research methodology ^33^^,^^34^.

### Statistical analysis

The interrater reliability of the researchers who undertook the scoring process using the Quality List and the JBI-MAStARI control list was analyzed using the SPSS 22.0 software and the kappa statistic. Descriptive statistics are presented separately for each reviewed study as frequencies (N), percentages (%), and standard deviation (SD) values.

## Results

Figure 1 shows a flowchart summarizing the inclusion process of the studies in this systematic review. A total of 907 studies were found in the database search. Of these studies, 205 were eliminated due to duplication, and the remaining 702 were analyzed. As a result, 686 were eliminated based on their abstracts/titles and full-text according to the PICOS framework and inclusion criteria. Three studies were not included in the review as they were considered to have poor quality.

### Characteristics of the included studies

The systematic review included 13 studies. The sample sizes of these studies varied between 44 and 3 235 infants. Ten of the studies (76.9%) were conducted in Asian countries (India, Turkey, Bangladesh, and Indonesia), two (15.3%) were conducted in African countries (Cameroon and Kenya) and one (7.6%) was conducted in the USA.

As a result of the analyses of the study designs: we found that five studies were cross-sectional (38.4%), three were descriptive (23%), two (15.3%) were quasi-experimental, and the remaining were case-control, and cohort studies (7.6% each). An e-mail was sent to the author of one of the studies for additional information ^35^.

The examined studies reported that babies were born at term with birth weights varying between 2.7 and 3.2 kg. The mean age of mothers ranged between 18 and 29.5 years. Seven studies reported the percentages of water intake in infants (%) ^11^^-^^13^^,^^26^^,^^36^^,^^37^^,^^38^ two studies reported the volumes of daily water consumption ^27^^,^^35^, one study indicated the volume of water consumption in the last 24 hours ^38^, and five studies showed the time of the first water intake ^27^^,^^28^^,^^39^^,^^40^^,^^41^. One study reported the properties of the consumed water ^27^, and only one study clearly stated the reasons for water intake ^27^.

The characteristics of the studies included in this systematic review are shown in Table 1.

**Table 1.** Articles included in the systematic review

Authors/ Year/ Country	Design/ sample size N/n*	Aim of the research	Socio-demographic characteristics of the infant	Socio-demographic characteristics of the family	Infants’ feeding patterns (0-6 months)	Properties of water	Effective factors	Starting age of water intake (0-6 months)	Frequency and amount of water consumption (0-6 months old)	Reasons for water consumption in infants (0-6 months old)	S Stduies Quality Assessment
Yüzügüllü *et al*., (2018) ^11^, Turkey	Cross sectional studies/ N=284	This study was conducted in Çukurova county of Adana province To investigate mothers’ sociodemographic and psychopathological characteristics that affect their exclusive breastfeeding status for the first six months.	Gender % (n): Male: 50 (142) Female: 50 (142)	Mother’s age % (n): ≤19 years: 1,1 (3) 20-24 years: 11,6 (33) 25-29 years: 23,9 (68) 30-34 years: 40,5 (115) 35-39 years: 16,5 (47) ≥40 years: 6,3 (18) Mother's educational status % (n): Illiterate: 0,4 (1) Literate: 0,4 (1) Primary school: 11,3 (32) Middle school: 27,8 (79) High school: 27,8(79) College/university: 32,4 (92) Mother’s working status % (n) Working: 18,3 (52) Not working: 68,0 (193) Working but on leave: 13,7 (39)	Breast milk: 37 % Formula: 48,7 %	-		-	The rate of those who give breast milk and water in the first six months is 2.5 %.		5/8
Özgürhan *et al*. (2020) ^12^/ Turkey	Descriptive, prospective/ n:355	The aim of this study was to examine the factors leading mothers to discontinue exclusive breastfeeding within the first six months of life.	Gender (male/ female) (n) 175/180 Height (cm) (mean ± SD) 50.1±1.2 Weight (g) (mean ± SD) 3201.4±469.2 Head circumference (cm) (mean ± SD) 34.9±0.8	Mother Age (years) (mean ± SD) 28.3±5.2 Delivery method Vaginal/cesarean section (n) 179/176	Breastfeeding at 1-month-old: 68.4 %; Breastfeeding at 4-months-old: 56.5 % Breastfeeding at 6 months-:35.2 %				six months: 26 (7.3 %)		8/9
Susanto *et al* (2021) ^13^/ Indonesıa	Cross-sectional study N: 470 n: 470	Maternal and child health status (MCHS) plays an essential role in the exclusive breastfeeding practice (EBP), which in turn helps to determine the nutritional status and development of children (NSC & CD) aged 0-6 months. This study aimed to determine the prevalence and factors influencing EBP, NSC, and CD to MCHS in Jember, East Java, Indonesia.	Characteristics of the children Age(months) M±SD 3.91±1.63 Gender n (%) Boys 255 (54.3) Girls 215 (45.7) Weight of birth (gram) M±SD 062.90±459.78 Height of birth (cm) M±SD 49.04±2.71	Characteristics of the mothers Age (years) M±SD 22.71±6.22 Education Not attending schools 13 (2.8) Elementary schools 141 (30.0) Junior high schools. 118 (25.1) Senior high schools. 151 (32.1) Diploma-3 7 (1.5) Bachelor 40 (8.5) Is the mother working? No 382 (81.3) Yes 88 (18.7) The family income per month (IDR) M±SD. 1547978.72±1172358.97 Parity status Primipara 170 (36.2) Multipara 300 (63.8) Type of family Elementary family 216 (46.0) Extended family 254 (54.0)	Exclusive breastfeeding 351 (74.7) No exclusive 119 (25.3)	-		-	Pre-lacteal feeds given n (%) (n= 126) Plain boiled water 3 (2.4) Post-lacteal feeds given n (%) (n= 124) Plain boiled water 3 (2.5)		6/8
Oiye *et al*., (2017) ^26^, Kenya	Quase-Experimental Studie** N= 143 n= 68	The study aimed to compare exclusive breastfeeding practice between HIV-positive versus HIV-negative mothers using the maternal recall method and DO technique.	-Healthy babies -Infants with birth weight inferior to 2,500 g Gender (n): Male: 37 Female: 31	Age of the mother (years) (mean ± SD): 25.6±6.4 Attended secondary school or above (n): 21 Living in the rural areas (n): 42	Breast milk Supplementary food Exclusive breastfeeding rate maternal recall (DO technique): Six weeks: 76.9 % (13.8 %) 6 months: 59. % (24.2 %) Bias: 41 (63.1 %)	-		-	DO technique: Six weeks: 86.2 % Six months: 75.8 %	-	7/9
Demir *et al*. (2020) ^27^/ Turkey	Descriptive/ N: 187	This study was planned to determine the breastfeeding status of the mothers who applied to family health centers (FHC) and who have 0-24 months old baby and child, the feature of giving water to their baby, the time to start giving regular water, and what type of water they prefer to give their child.	Median months of infants and children: 11.0 (5.0-20.0) 0-24 months-old babies	The mean age of 187 mothers participating in the study was 29.1±5.0 years, 65.2 % of the mothers stated they had a high level of education, 63.1 % did not work, and 84.5 % stated that their income level was not high.		Mothers with low education levels; -tap water 21 (33.9 %), -packaged water 13 (21.0 %) -boiled water 28 (45.2 %) Mothers with a high level of education; -tap water 21 (19.3 %), -packaged water 16 (14.7 %) -boiled water 72 (66.1 %) Of the mothers, 58.5 % stated that they preferred to give boiled water when giving water to their babies for the first time. Also, 62.6 % of the participants stated that they never gave tap water to their children, and mothers who gave tap water to their children stated that they gave it to their babies for the first time when they were 8.0 (5.0-11.0) months old.	The rate of giving water in the first six months was found to be higher in mothers with lower education levels than in mothers with higher education levels (p=0.001). Those with higher education levels stated that they mostly preferred boiled water when giving water to their baby for the first time (p = 0.026). It has been observed that working mothers have lower rates of giving water to their babies in the first six months than non-working mothers. A statistically significant difference was found between the income levels of the mothers and the status of giving tap water to their babies. (p = 0.020).	Of the mothers, 62.6% stated that they gave water to their babies in the first six months and 14.5 % of them reported they gave sweetened water to their babies in the first days. Also, 34.2 % of the participants stated that they gave water to their babies for reasons other than the transition to complementary foods or formula supplements. Mothers stated that they started giving water to their babies regularly when they were 5.0 (2.0-6.0) months old.	Included infants aged six months and younger were found to consume 100.0 (50.0-100.0) mL of water within 24 hours.	First reason of the non-working mothers for giving water to the infants -Complementary food/formula supplement 56 (52.8 %) -Other 50 (47.2 %) First reason of working mothers for giving water -Complementary food/formula supplement 51 (78.5 %) -Other 14 (21.5%) In addition, 34.2 % of the participants stated that they gave water to their babies for reasons other than the transition to complementary foods or formula supplements.	(5/9
Yılmaz (2019) ^28^/ Turkey	Descriptive /N:164	This study aimed to examine the feeding patterns of 0-24-month-old infants hospitalized in the infant service of a state hospital.	0-24-months-old healthy babies Gender: Girl: 65 (39.6 %) Boy: 99 (60.4 %) Month: 0-6: 61 (37.2 %)	Average age of the mothers: 26.82 ± 5.47 Education level of the mother Primary school 79 (48.2 %) Middle School 30 (18.3 %) High School 13 (7.9 %) Literate 32 (19.5 %) Illiterate 10 (6.1 %) Mother’s Job Housewife: 164 (100 %) Family type Elementary family: 111 (67.7 %) Family type: 53 (32.3 %) Perceived income status Bad 126 (76.8 %) Medium 34 (20.8 %) Good 4 (2.4 %)	0-6 month Exclusive breastfeeding: 18 (29, 5 %) Breastfeeding + water: 14 (23,0 %) Breastfeeding + Formula feeding: 20 (32,7 %) Breastfeeding + complementary feeding: 5 (8,2 %) Breastfeeding + Formula feeding + complementary feeding: 2 (3,3 %) Other: 2 (3,3 %)				Before six months: 116 (70.7 %)		6/9
Medoua *et al*. (2012) ^35^/ Kamerun	Cross-sectional/ n:44	The present study measured breast milk and non-breast milk water intake using the dose-to-the-mother deuterium-oxide turnover technique to validate mothers’ reports of infant feeding practices.	Age of the infant (month): 2.7 ± 1.3 Infant’s birthweight (kg): 3.2 ± 0.6 Infant’s weight (kg): 6.1± 1.4 Infant’s length (cm): 56.0 ± 5.0 Gender ratio (male/female): 15/29	Age of the mother (years):26.6 ± 5.1 Mother’s BMI (kg m2-1 ): 26.8 ± 3.7	*The statements of the mothers were confirmed by the deuterium oxide technique. Expression of mothers and deuterium oxide results were given separately. The bias difference between the two data was also reported. Dietary recall since birth % (n) Exclusive breastfeeding (EBF): 45.4 % (20) Predominantly breastfeeding (PreBF): 15.9 % (7) Partial breastfeeding (ParBF): 38.6 % (17) Dose-to-the-mother deuterium-oxide turnover % (n) Exclusive breastfed (EBF): 11.4 % (5) Bias 34.06 % (15 ) Predominantly breastfed (PreBF): 45.4 % (20) Bias: -29.5 (13) Partially breastf ed (ParBF): 43.2 % (19) Bias: -4.6 (2)	-		-	Non-breast milk water intake (mL day-1 ) EBF:24.0 ± 13.4 PreBF:113.5 ± 41.8 ParBF:495.4 ± 223.9 Total:268.2 ± 250.3		6/8
Onbaşı *et al*., 2011 ^36^/ Turkey	Quasi-experimental study Intervention group (N:90) Control group (N:100)	Inform expectant mothers about breast-milk and breast-feeding via prenatal training and emphasize the advantages of the training.	Intervention group Birth weight 3,134 ± 613 Gender (Female) 56.7 % Type of birth 28.9 % Vaginal Control group Gender 56 % (Female) Birth weight 3,113 ±6 20 Type of birth 32 % Vaginal	Intervention group Mother’s average age n (%) 18-24 years 17 (18.9) 25-34 years 61 (67.8) 35 years and older 12 (13.3) Mother's educational status n (%) Illiterate 1 (1.1) Literate 1 (1.1) Primary school 35 (38.9) Middle School 13 (14.4) High school 22 (24.4) University 18 (20.0) Job n (%) Housewife 58 (64.4) Working 32 (35.6) Family structure n (%) Elementary family 79 (87.8) Extended family 11 (12.2) Control group; Mother age n (%) 18-24 years 15 (15.0) 25-34 years 64 (64.0) 35 years and over 21 (21.0) Mother's educational status n (%) Illiterate 4 (4.0) literate 1 (1.0) Primary school 30 (30.0) Middle School 17 (17.0) High school 27 (27.0) University 21 (21.0) Job n (%) Housewife 63 (63.0) Working 37 (37.0) Family structure n (%) Elementary family 82 (82.0) Extended family 18 (18.0)	Intervention group Only breastfeeding n(%): 6 months: 61 (67.8) Less than six months: 29 (32.2) Mean: 4.9 ± 1.8 Formula food/ complementary food use n (%): 29 (32.2) Control group Only breastfeeding n(%): 6 months: 28 (28.0) Less than six months: 72 (72.0) Mean: 3.2±2.4 Formula food/ complementary food use n (%): 72 (72.0)	-			Intervention group Water n (%) 25 (27.8) Control group Water n (%) 50 (50.0)	-	7/9
Kay *et al* (2018) ^37^/ US	Cross-Sectional Study N:3235 (n = 600)	The study used data from the Feeding Infants and Toddlers Study 2016 to describe the beverage consumption patterns of infants and young children (0-47.9 months)	Gender (%) Female: 50 % Male: 50 % Child’s race/ ethnicity (%) Hispanic: 16 White (non- Hispanic): 65 Black (non- Hispanic): 13 Other (non- Hispanic): 6.3	Mother's education level (%) Less than a high school diploma or GED 4.1 Completed high school or GED 19 Some college/postsecondary 28 Completed college 38 Some graduate work/ degree 11 Household income level (%) Less than $10,000 9.3 $10,000 to $19,999 9.3 $20,000 to $34,999 18 $35,000 to $49,999 17 $50,000 to $74,999 20 $75,000 to $99,999 14 $100,000 to $149,999 8. 3 $150,000 or more 3.7	Nutritional Pattern of the Infant: % (± SE) Breast milk 53.5 (2.8) Infant formula 62.2 (2.6) Whole milk 1,4 (0.6) Reduced fat milk 0.2 (0.2) Low-fat milk 0.2 (0,2) Non-fat milk 0.1 (0.1)	-		-	Water % (± SE) 10.0% (1.5)	-	5/8
Islam *et. al*. (2014) ^38^/ Bangladesh	Cross-Sectional Study/ N: 120 n:29	The study aimed to detect the amount of arsenic in the human milk of lactating mothers and relate this with maternal and children’s urinary arsenic in arsenic-contaminated areas in Bangladesh.	Child’s gender, n (%): Male: 15 (51.7 %) Female 14 (48.3 %) Child’s birth weight in kg: 3.2 ± 0.6	Mother’s average age: 24.6 ± 5.0 Religion Muslim: 19 (65.5) Hindu: 10 (34.5) Number of pregnancies: 2.5 ± 1.7 Number of live births: 2.4 ± 1.6	Food given in the past 24 hours at six months: Breast milk: 26 (89.7)				Findings indicated that 96.5 % of 6-month-old babies were given water in the last 24 hours.		6/8
Geçkil *et al*. (2012) ^39^/ Turkey	Quasi-experimental Intervention group: N:42 Control group: N:52	This study was conducted to examine the effect of the "Breastfeeding Support and Monitoring Program" implemented by Family Health Staff on effective breastfeeding behaviors of mothers in the first six months after birth.	Month: Healthy infants under six months Gender Intervention: Girl: 47.6 % Boy:52.4 % Control Girl: 57.7 % Boy: 42.35 %	Intervention group; Mother’s average age: 29.52 ± 5.71 Mother’s level of education Primary School Graduate 24 (57.2 %) Graduate Of Secondary School 9 (21.4 %) High school and graduate 5 (11.9 %) Illiterate 4 (9.5 %) Mothers with health problems Yes 7 (16.7 %) No 35 (83.3 %) Control group Average mother age: 28.03 ± 5.30 Mother’s level of education Primary School Graduate 31 (59.6 %) Middle School 5 (9.6 %) High school and graduate 9 (17.3 %) Illiterate 7 (13.5 %) Mother's health problem Yes: 9 (17.3 %) No: 43 (82.7 %)	Intervention group Time to start complementary feeding: 5.81 ± 0.98 (n=38) Control group Time to start complementary feeding: 5.27 ± 1.51 (n=43)			Intervention group 5.04 ± 1.34 (n=42) Control group 2.78 ± 1.70 (n=47) (t=-6.888, p<0.001)			6/9
Samuel *et al*., (2012) ^40^, India	Cohort study/ n=50	This study was designed to measure rates of breastfeeding in infants born in a baby-friendly hospital in Bangalore, India, and to capture home based compliance to exclusive breastfeeding.	Body weight (kg) (mean ± SD): At delivery/birth: 2.7 ± 0.5 Month 1: 3.8 ± 0.6 Month 3: 5.6 ± 0.8 Month 6: 7.2 ± 1.0 Body length (cm) (mean ± SD): At delivery/birth: 49.6 ± 1.7 Month one: 53.2 ± 2.4 Month three: 60.8 ± 2.8 Month six: 65.8 ± 2.7	Age of the mother (years) (mean ± SD): 23.0 ± 2.9 Parity (n): Primiparous: 38 Multiparous: 12 Educational level (n): Up to high school (10th grade): 24 12th grade and above: 26 Employment status (n): Employed outside home: 9 Homemaker: 41 Monthly household income (INR) (mean): 9000	Breast milk, formula, and complementary feeding Mothers reported starting complementary foods (Infants exclusively breastfed were assessed by deuterium dilution method): Month one: 0 (56 %) Month three: 1 % (22 %) Month six: 64 % (14 %)	-		Month one	Deuterium Oxide technique: Month 1: 44% Month 3: 77% Month 6: 85% Intake of non-breastmilk water intake significantly increased from months one to six (p<0.01).	-	8/11
Yılmaz *et al*. (2022)* ^41^/Turkey	Case/control Control group N:60	This study aimed to investigate the effect of dietary factors on those diagnosed with “idiopathic” infantile urolithiasis.	Control Group month: 8.7 (2-24) Gestational age at delivery: term birth Control groups: Female/male: 23/37	-	Control group: 29 (48 %) patients were breastfed exclusively.	-		Control group: 4.3 (1-6) months	-		8/10

* Since the babies of the study group were diagnosed with urolithiasis, the control group was written.

** The water consumption of infants of HIV-negative mothers was examined.

N: sample in the article

n: number of 0-6 month-old babies in the sample

### Quality of included studies

One of the articles included in the systematic review ^40^ was evaluated with the cohort studies quality list, one ^41^ was assessed with the case- control studies checklist, five ^11^^,^^13^^,^^35^^,^^37^^,^^38^ were evaluated with the cross-sectional studies checklist, three ^12^^,^^27^^,^^28^ were analyzed with the descriptive studies checklist, and three ^26^^,^^36^^,^^39^ were assessed with the quasi-experimental studies checklist (table 1).

Since the quality of the evidence in the articles was considered medium or high, according to the requirements of the mentioned checklists, these were included in the systematic review. The cohort study met eight [40] of the eleven criteria in the checklist, the case-control study met eight ^41^ out of the ten, the cross-sectional studies met five ^11^^,^^37^, and six ^13^^,^^35^^,^^38^ of the eight criteria, the descriptive studies met five ^27^, six ^28^, and eight ^12^ out of the nine, and the quasiexperimental studies met six ^39^, and seven ^26^^,^^36^ of the nine criteria.

The general quality scores of the studies ranged between 55 to 88%. Additionally, the kappa value calculated to determine the interrater agreement for the JBI quality assessment tools was 0.66 for the experimental and quasi- experimental studies checklists, 0.87 for the descriptive studies checklist, 0.79 for the cohort studies checklist, 0.61 for the case-control studies checklist, and 0.72 for the cross-sectional studies checklist. Accordingly, a high level of interrater agreement was achieved ^42^.

### Nutritional status of infants

In the studies included in the systematic review, the rate of exclusive breastfeeding in infants, aged 0-6 monthsold, ranged between 24.2%, and 74.7%, and the rate of feeding with formula varied between 11% and 62. %. The rate of breastfeeding and formula feeding together was 41% in one study. The nutritional status of infants was not reported in two studies ^38^^,^^39^.

### Water consumption in infants

Details about the water consumption of 0-6-month-old babies included the percentage of water intake (%), the volume of daily water consumption, the volume of water consumption in the last 24 hours, and the time of the first intake. The rates of water intake in infants were reported as 44% in 1 -month- olds ^40^, 86.2% in 6 weeks-olds ^26^, 77% in infants aging 3-months-old ^40^, 2.5% in infants aging 4months-old ^13^, and 2.5-85% in infants aging 6-months-old ^11^^,^^12^^,^^26^^,^^36^^,^^40^. Moreover, the studies reported that 10% ^37^ and 96.5% ^38^ of the 6-month-old babies consumed water in the last 24 hours. The volumes of daily water consumption were 268.2 ± 250.3 ml/day in 1-3-month-old babies ^40^, and 100-268 ml/day in 6month-old and younger babies in one study ^27^^,^^35^. The average time of the babies’ first water intake was 1-5.04 months old ^27^^,^^39^^,^^40^^,^^41^. Seventy-point-seven percent (70.7%) of mothers started giving water before the sixth month ^28^, and some others started giving water to their infants in the first month ^40^.

### Properties of the consumed water by infants

Demir *et al*. (2020) ^27^ stated that mothers with low levels of education used tap water (33.9%), bottled water (21.0%), and boiled water (45.2%), and mothers with high levels of education gave their babies tap water (19.3%), bottled water (14.7%), and boiled water (66.1%).

### Reasons for giving water to infants

Reasons for giving water to babies included the transition to complementary food/formula (52.8-78.5%) and other reasons (21.5-47.2%) ^27^.

### Water consumption effective factors

The rate of giving water to infants, in their first six months, was higher for mothers with lower education levels than for mothers with higher education levels (p=0.001). Those with higher education levels preferred boiled water for the first water intake of their infants (p=0.026) ^27^.

Working mothers have lower rates of giving water to their babies in their first six months of life than non-working mothers. A statistically significant difference was found between the low-income levels of the mothers and giving tap water to their babies (p=0.020) ^27^.

## Discussion

This systematic review determined the water consumption of 0-6-month- old infants and the factors affecting this consumption. The findings of this study are discussed here in line with the relevant literature.

### Water consumption in infants

In this systematic review, the rates of 0-6-month-old babies fed with water in addition to breastmilk were between 2.5% and 85% ^11^^,^^12^^,^^26^^-^^28^^,^^36^^,^^40^. In the 24hour follow-ups of 6-month-old babies, the water consumption in one study was 96.5% ^38^ and in another 10% ^37^; for the 3-month-old infants, this consumption was 77% ^40^. In a study with babies younger than six months, threequarters of caregivers ^43^ and 52.9% of the mothers ^44^ gave water to their babies in the first six months ^43^^,^^44^.

The WHO (2022) actively promotes breastmilk as the best source of nutrition for infants and toddlers and aims to increase the rate of exclusive breastfeeding to at least 50 % during the first six months by 2025 ^45^. Different studies have emphasized that giving water to babies within the first six months of the postpartum period could be one of the reasons for the cessation of breastfeeding in the early period and the reduced interest of the babies in breastmilk ^37^^,^^46^^,^^47^.

This situation is a barrier to achieving 2025 WHO’s goals. In a Cochrane systematic review examining early supplementary food and fluid intake in healthy and breastfed term infants, the rate of continued breastfeeding in exclusively breastfed infants was higher than that of infants drinking water/ sweetened water in their first days after birth ^17^. A randomized controlled trial about decreasing water and herbal tea intake in breastfed infants in the first six months of life reported that 35% of infants in the control group stopped breastfeeding due to water and herbal tea intake ^48^.

In the reviewed literature, most studies did not specify the amount of water intake in babies during their first six months. However, the findings of our systematic review showed that infants consumed an average of 100 (50.0- 100.0) to 268.2 ± 250.3 mL of water in their first six months ^27^^,^^35^. Similarly, Grimes *et al*. (2017) stated that 05.9-month-old babies consumed 15 ± 2 ml of water per day ^49^. Is possible that the difference between the reported amounts is the starting moment of water consumption and complementary foods in babies. Health professionals should emphasize the appropriate age and timely introduction of supplemental water and complementary foods in infant nutrition education. An excess of water can not replace human milk as feeding, resulting in a lower intake of nutrients and premature termination of exclusive breastfeeding ^38^.

In the studies included in this systematic review, the average time of the first water consumption in babies was 1-5.04 months old in general ^27^^,^^39^^,^^40^^,^^41^. Onah *et al*. (2014) found that 22.3% of their participants gave water to their babies as the first food to be consumed ^46^. Khatun *et al*. (2018) stated that more than half of mothers gave different foods to their babies in the first three days of life, and water made up 10.8% of the additional foods given to babies ^43^. In another study, it was revealed that 38.9% of mothers started giving water to their babies in their first month ^50^. One study examined the first baby’s water intake in four different stages: 47.8% of the participants gave water to their infants right after birth, 12.47% gave it when their babies were 1 -2 months old, 14.7% gave it when their babies were 3-4 months old, and 25% gave it when their babies were 5-6 months old ^44^. In line with the recommendations of reliable health authorities, these studies showed that exclusive breastfeeding rates in the first six months are low. The rates of exclusive breastfeeding in mothers who first fed with breast milk their newborns were 3.36 and 6.22 times higher than the rates of the mothers who first fed their infants with water/water-based solution and formula respectively ^46^.

In the studies included in this systematic review, it was not clear who decided to give water to the infants, but in any case, this indication should be made by health professionals. New studies are needed to reveal these issues. Water intake in infants younger than six months old may result in the premature termination of nutrient-rich human milk consumption which can lead to troubling consequences ^25^. Martins and Giugliani (2012) found that late intake of water, tea, and other types of milk to babies increased the likelihood of continued breastfeeding for two years or longer ^51^. With regular counseling sessions for adolescent mothers, which started in the maternity ward and continued for the first four months, it was possible to reduce the discontinuation rate of exclusive breastfeeding by 52%, avoiding unnecessary water or tea intake ^48^.

### Properties of the consumed water

The types of water used in the studies included in this systematic review were classified as tap, boiled, and packaged ^27^. One study indicated that water specifically produced for babies was preferred in the first four months ^50^, while another one reported that tap water and bottled water were preferred among the participants ^49^. A study conducted in Nigeria stated that the most common types of drinking water included river/lake bed water, especially well water, borehole water, and tap water ^44^. Demir *et al*. (2020) associated the water preferences of mothers with their education levels, mothers with lower education levels preferred tap water, while mothers with higher education levels preferred boiled water ^27^.

Even the consumption of small volumes of water or other nutrients in addition to human milk during the first six months may increase the risks of diarrhea and infections ^35^^,^^44^^,^^50^. Nutritional practices, vital for Infants and children, are an important factor in the prevention of parasitic infections. The longer 0-6-month-old infants are breastfed, the more they will be protected from exposure to contaminated water and low-nutrient complementary foods prepared with water. High-quality nutrition for the child in the first six months will increase their immunity and resistance to pathogens ^52^. Health professionals should encourage mothers to prefer safe and inexpensive water from breast milk for their babies ^27^.

### Reasons for giving water to infants

Among the studies included in this systematic review, only one study was found to mention reasons for giving water to babies, which included the transition to supplementary food/formula and other reasons, but these reasons were not clearly stated ^27^. In other studies, the prominent reasons for giving water to infants included the thought that the infant was thirsty ^43^^,^^44^^,^^50^ and cultural reasons ^53^^,^^54^. In some studies, it was observed that mothers misunderstood the concept of exclusive breastfeeding. They thought water could be given as long as they only feed the infant with human milk ^47^^,^^54^. In another study, it was stated that although 82% of mothers knew about exclusive breastfeeding, the rate of compliance with this recommendation was 33.5%. This finding explained why having exclusive breastfeeding knowledge does not necessarily turn it into a practice ^55^. In the study conducted by Vygen *et al*. (2013), one mother stated that mothers were taught about exclusive breastfeeding in the antenatal clinics of the health center, but they did not comply with it. She thought it would be unfair to deprive the infant of water while she was drinking it. She felt like she was punishing her infant ^53^.

The results of this study are important for nurses and other health professionals. The visits of the infant to the hospital for vaccination, health follow-ups, and growth follow-ups should be considered critical interaction points of the mother with nurses and other healthcare professionals for emphasizing the importance of exclusive breastfeeding ^40^. Nurses and other healthcare professionals should reconsider their approach to breastfeeding starting from pregnancy and provide mothers with scientific evidence on reasons to avoid giving water to their babies.

Some studies reported the presence of social pressure and the influence of grandmothers on giving water to the infant in the early period ^54^^,^^55^. In a study with grandmothers conducted by Ferreira *et al*. (2018), the participants emphasized the importance of giving water to babies in the first days of life to calm them because they thought human milk did not quench thirst ^54^.

### Water consumption effective factors

An included study showed that the education level, employment status, and family income level of the mothers influenced the practice of giving water to their babies in the first 6 months. Mothers with low education levels, not working, and low family income gave water to their babies in the first six months ^27^. In some studies, drinking water preferences of mothers involved many factors such as taste, smell, hardness, quality, advertising, marketing, and accessibility to drinking water resources; other studies considered socio-demographic characteristics such as age, gender, education level, and income level ^56^^,^^57^. Mothers with a higher level of education are more likely to have more information about infant nutrition than mothers with lower levels of education, which are usually more attached to traditions.

The results of this systematic review are important because they revealed the points to be emphasized in infant nutrition education programs by nurses and other health professionals. While deciding about breastfeeding, mothers may often feel obligated to choose between the recommendations of health authorities and the recommendations of elders, society, or traditions. Design of interventions that target not only mothers but also family elders and society can help to identify and overcome barriers to exclusive breastfeeding in the first six months. The results of this systematic review showed that more studies with high levels of evidence are needed on this topic.

Although this study had several strengths, it also had some limitations. Most of the articles included in this systematic review did not adequately explain the reasons for giving water to infants, the properties of the consumed water, and the factors affecting the introduction of water into the infant’s diet. In addition, three studies included in the systematic review are supported by the use of the deuterium-oxide technique to confirm mothers’ statements.

The results of the research found lower levels of breast milk intake than those declared by the mothers. Other studies included were based only on the mothers’ statements. Additionally, in the selected articles was insufficient data on whether water was introduced based on the recommendation of a health professional as a result of a medical indication or for other reasons.

Reported rates of water consumption in infants aged 0-6 months varied between 2.5 and 70.7%. Liquids given to infants included tap, boiled, bottled, packaged, sweetened, carbonated, enriched, fortified, and infant water. It was noted that the most striking factors related to reasons for giving fluids to infants were concerns about infant dehydration and the intense influence of family members.

In line with the recommendations of reliable health authorities, it is necessary to disseminate the information that 0-6-month-old babies do not need water and other fluids when they are exclusively breastfed. We recommend providing families with up-to-date evidence on this issue starting from pregnancy. It is important to increase the quality of evidence by conducting randomized controlled trials in different cultures that will provide up-to-date information on the subject. Efforts to inform families about not interfering with natural nutrition without consulting health professionals should be supported.

## References

[1] Şatır G, Çelik M, Kemhacıoğlu M. (2017). Infant feeding practices of breast-feeding mothers and affecting factors. Med J SDU.

[2] Sinno D, Tamim H, Faytrouni F, Mikati MA, Charafeddine L (2018). Factors affecting child development assessed by the Ages and Stages Questionnaire (ASQ) in an Arabic speaking population. Early Human Development.

[3] General Assembly, United Nations (UNGA) (2015). Transforming our world: The 2030 agenda for sustainable development.

[4] World Health Organization (WHO) (2017). Protecting, promoting and supporting breastfeedıng in facilities providing maternity and newborn services.

[5] United Nations International Children’s Emergency Fund (UNICEF) (2018). Breastfeeding from the first hour of birth: what works and what hurts.

[6] World Health Organization (WHO) (2014). United Nations International Children’s Emergency Fund (WHO/UNICEF).

[7] United Nations International Children’s Emergency Fund (UNICEF) (2018). “Infant and young child feeding. Percent of infants 0-5 months of age exclusively breastfed, by country and UNICEF region, 2018”.

[8] Victora CG, Bahl R, Barros AJ, França GV, Horton S, Krasevec J (2016). Lancet Breastfeeding Series Group. Breastfeeding in the 21st century: epidemiology, mechanisms, and lifelong effect. Lancet.

[9] Irmak N. (2016). The importance of breastmilk and the factors that affect exclusive breastfeeding. Jour Turk Fam Phy.

[10] (2018). Turkey Demographic and Health Survey (TNSA).

[11] Yüzügüllü DA, Aytaç N, Akbaba M (2018). Investigation of the factors affecting mother’s exclusive breastfeeding for six months. Turk Pediatri Arsivi.

[12] Özgürhan G, Cömert S (2020). An evaluation of the factors affecting exclusive breastfeeding. Istanbul Medical Journal.

[13] Susanto T, Yunanto RA, Rasni H, Susumaningrum LA (2021). Maternal and child health status related to nutritional status and development of children during lactation period: A cross- sectional study among mothers with children age 0-6 months In Agricultural Areas of Indonesia. Malaysian Journal of Public Health Medicine.

[14] Gün İ, Yılmaz M, Şahin H, İnanc N, Aykut M, Günay O (2009). The breast-feeding status of 0-36-month-old children in Kayseri, Melikgazi education and research area. Journal of Child Health and Diseases.

[15] Unger M. (2020). Barriers to fully informed decisions on whether to breastfeed or formula feed in the United States. Hastings Women’s.

[16] Tetik KB. (2016). Current information on consultancy of breast milk and breast-feeding. Ankara Med J.

[17] Smith HA, Becker GE (2016). Early additional food and fluids for healthy breastfed full-term infants. The Cochrane Database of Systematic Reviews.

[18] Leghi GE, Middleton PF, Muhlhausler BS (2018). A methodological approach to identify the most reliable human milk collection method for compositional analysis: a systematic review protocol. Systematic Reviews.

[19] Heinig MJ, Nommsen LA, Peerson JM, Lonnerdal B, Dewey KG (1993). Intake and growth of breast-fed and formula-fed infants in relation to the timing of introduction of complementary foods: the DARLING study. Davis Area Research on Lactation, Infant Nutrition and Growth. Acta Paediatrica.

[20] Glover J, Sandilands M (1990). Supplementation of breastfeeding infants and weight loss in hospital. Journal of Human Lactation.

[21] De Carvalho M, Hall M, Harvey D (1981). Effects of water supplementation on physiological jaundice in breast-fed babies. Archives of Disease in Childhood.

[22] Yamauchi Y, Yamanouchi I (1990). Breast-feeding frequency during the first 24 hours after birth in full-term neonates. Pediatrics.

[23] Houston MJ, Howie PW, McNeilly AS (1984). The effect of extra fluid intake by breast fed babies in hospital on the duration of breastfeeding. Journal of Reproductive and Infant Psychology.

[24] Nicoll A, Ginsburg R, Tripp JH (1982). Supplementary feeding and jaundice in newborns. Acta Paediatrica Scandinavica.

[25] Demmer E, Cifelli CJ, Houchins JA, Fulgoni VL (2018). 3rd Ethnic disparities of beverage consumption in infants and children 0-5 years of age; National Health and Nutrition Examination Survey 2011 to 2014. Nutrition Journal.

[26] Oiye S, Mwanda W, Mugambi M, Filteau S, Owino V (2017). Exclusive breastfeeding is more common among hiv-infected than hiv-uninfected kenyan mothers at 6 weeks and 6 months postpartum. Breastfeeding Medicine.

[27] Demir LS, Eren G, Santa§ T, Uyar M, Durduran Y, §ahin TK (2020). Drinking water usage preferences of women who apply to family health centers in Meram district of Konya city center. Turkish Bulletin of Hygiene and Experimental Biology.

[28] Yilmaz G. (2019). Investigation of nutrition patterns of 0-24 months babies. Gümü§hane University Journal of Health Sciences.

[29] Topal S, Qinar N, Altinkaynak S (2016). Nutrition in infancy. Journal of Duzce University Health Sciences Institute.

[30] Page MJ, McKenzie JE, Bossuyt PM, Boutron I, Hoffmann TC, Mulrow CD (2021). The PRISMA 2020 statement: an updated guideline for reporting systematic reviews. BMJ.

[31] Munn Z, Tufanaru C, Aromataris E (2014). JBI’s Systematic Reviews: Data Extraction and Synthesis. AJN The American Journal of Nursing.

[32] Nahcivan N, Secginli S (2017). How are the methodological quality of quantitative studies included in systematic reviews?. Turkiye Klinikleri J Public Health Nurs-Special Topics.

[33] Moola S, Munn Z, Tufanaru C, Aromataris E, Sears K, Sfetcu R, Currie M, Qureshi R, Mattis P, Lisy K, Mu P-F, Aromataris E, Munn Z (2020). JBI Manual for Evidence Synthesis.

[34] Tufanaru C, Munn Z, Aromataris E, Campbell J, Hopp L, Aromataris E, Munn Z (2020). JBI Manual for Evidence Synthesis.

[35] Medoua GN, Sajo Nana EC, Ndzana AC, Makamto CS, Etame LS, Rikong HA (2012). Breastfeeding practices of Cameroonian mothers determined by dietary recall since birth and the dose-to-the-mother deuterium-oxide turnover technique. Maternal & Child Nutrition.

[36] Onbaşı Ş, Duran R, Çiftdemir NA, Vatansever Ü, Acunaş B, Süt N (2011). The effect of prenatal breast-feeding and breast-milktraining given to expectant mothers on the behaviour of breast-feeding. Turk Arch Ped.

[37] Kay MC, Welker EB, Jacquier EF, Story MT (2018). Beverage consumption patterns among infants and young children (0-47.9 months): Data from the Feeding Infants and Toddlers Study. Nutrients.

[38] Islam MR, Attia J, Alauddin M, McEvoy M, McElduff P, Slater C (2014). Availability of arsenic in human milk in women and its correlation with arsenic in urine of breastfed children living in arsenic contaminated areas in Bangladesh. Environmental Health.

[39] Geçkil E, Şahin T, Tunçdemir A (2012). The effect of “the following and supporting breast-feeding programme”, that is applied by family health staff, on the mother’s effective breastfeeding behaviours in the first six months of the post-birth period. TAF Prev Med Bull.

[40] Samuel TM, Thomas T, Bhat S, Kurpad AV (2012). Are infants born in baby-friendly hospitals being exclusively breastfed until 6 months of age?. European Journal of Clinical Nutrition.

[41] Yilmaz AQ, Ünal N (2022). Do dietary factors play a role in infantile urolithiasis?. Pediatric Nephrology.

[42] Kilig S. (2015). Kappa Testi. Journal of Mood Disorders.

[43] Khatun H, Comins CA, Shah R, Munirul Islam M, Choudhury N, Ahmed T (2018). Uncovering the barriers to exclusive breastfeeding for mothers living in Dhaka’s slums: a mixed method study. International Breastfeeding Journal.

[44] Matthew AK, Amodu AD, Sani I, Solomon SD (2010). Infant feeding practices and nutritional status of children in North Western Nigeria. Asian Journal of Clinical Nutrition.

[45] World Health Organization (WHO) (2022). Breastfeeding. WHO Response.

[46] Onah S, Osuorah DI, Ebenebe J, Ezechukwu C, Ekwochi U, Ndukwu I (2014). Infant feeding practices and maternal socio-demographic factors that influence practice of exclusive breastfeeding among mothers in Nnewi South-East Nigeria: a cross-sectional and analytical study. International Breastfeeding Journal.

[47] Mogre V, Dery M, Gaa PK (2016). Knowledge, attitudes and determinants of exclusive breastfeeding practice among Ghanaian rural lactating mothers. Int Breastfeed J.

[48] Nunes LM, Giugliani ER, Santo LC, de Oliveira LD (2011). Reduction of unnecessary intake of water and herbal teas on breast-fed infants: a randomized clinical trial with adolescent mothers and grandmothers. The Journal of Adolescent Health.

[49] Grimes CA, Szymlek-Gay EA, Nicklas TA (2017). Beverage consumption among US children aged 0-24 months: National health and nutrition examination survey (NHANES). Nutrients.

[50] McLennan JD, Pérez Agramonte M, Mosquea HM (2022). A mixed method inquiry of early complementary feeding of infants in the Dominican Republic. Appetite.

[51] Martins EJ, Giugliani ER (2012). Which women breastfeed for 2 years or more?. Jornal de Pediatría.

[52] Palmieri JR, Meacham SL, Warehime J, Stokes SA, Ogle J, Leto D (2018). Relationships between the weaning period and the introduction of complementary foods in the transmission of gastrointestinal parasitic infections in children in Honduras. Research and Reports in Tropical Medicine.

[53] Vygen SB, Roberfroid D, Captier V, Kolsteren P (2013). Treatment of severe acute malnutrition in infants aged <6 months in Niger. The Journal of Pediatrics.

[54] Ferreira T, Piccioni LD, Queiroz P, Silva EM, Vale I (2018). Influence of grandmothers on exclusive breastfeeding: cross-sectional study. Einstein.

[55] Mundagowa PT, Chadambuka EM, Chimberengwa PT, Mukora-Mutseyekwa F (2019). Determinants of exclusive breastfeeding among mothers of infants aged 6 to 12 months in Gwanda District, Zimbabwe. International Breastfeeding Journal.

[56] Turner íkikat E, Birinci A, Yildirim Q (2011). Ambalajli su tüketimini etkileyen faktorlehn belirlenmesi: Ankara ili Kegióren ilgesi ornegi. Alinteri J Agrie Sci.

[57] Uzundumlu AS, Fakioglu Ó, Kóktürk M, Temel T (2016). Determining the best drinking water preference in Erzurum Province. Alinteri J Agrie Sci.

